# FEWheat-YOLO: A Lightweight Improved Algorithm for Wheat Spike Detection

**DOI:** 10.3390/plants14193058

**Published:** 2025-10-03

**Authors:** Hongxin Wu, Weimo Wu, Yufen Huang, Shaohua Liu, Yanlong Liu, Nannan Zhang, Xiao Zhang, Jie Chen

**Affiliations:** 1 College of Information Engineering, Tarim University, Alar 843300, China; 13183310868@163.com (H.W.); love_hyf@163.com (Y.H.); liu_5748688@163.com (S.L.); zhangnannan@taru.edu.cn (N.Z.); 2 Key Laboratory of Tarim Oasis Agriculture, Ministry of Education, Tarim University, Alar 843300, China; 3 Key Laboratory of Genetic Improvement and Efficient Production for Specialty Crops in Arid Southern Xinjiang, Alar 843300, China; wwmxjtlm@taru.edu.cn (W.W.); ylliu0817@163.com (Y.L.)

**Keywords:** wheat spike detection, YOLOv11n, lightweight deep learning model, small-scale wheat spike detection

## Abstract

Accurate detection and counting of wheat spikes are crucial for yield estimation and variety selection in precision agriculture. However, challenges such as complex field environments, morphological variations, and small target sizes hinder the performance of existing models in real-world applications. This study proposes FEWheat-YOLO, a lightweight and efficient detection framework optimized for deployment on agricultural edge devices. The architecture integrates four key modules: (1) FEMANet, a mixed aggregation feature enhancement network with Efficient Multi-scale Attention (EMA) for improved small-target representation; (2) BiAFA-FPN, a bidirectional asymmetric feature pyramid network for efficient multi-scale feature fusion; (3) ADown, an adaptive downsampling module that preserves structural details during resolution reduction; and (4) GSCDHead, a grouped shared convolution detection head for reduced parameters and computational cost. Evaluated on a hybrid dataset combining GWHD2021 and a self-collected field dataset, FEWheat-YOLO achieved a COCO-style AP of 51.11%, AP@50 of 89.8%, and AP scores of 18.1%, 50.5%, and 61.2% for small, medium, and large targets, respectively, with an average recall (AR) of 58.1%. In wheat spike counting tasks, the model achieved an R^2^ of 0.941, MAE of 3.46, and RMSE of 6.25, demonstrating high counting accuracy and robustness. The proposed model requires only 0.67 M parameters, 5.3 GFLOPs, and 1.6 MB of storage, while achieving an inference speed of 54 FPS. Compared to YOLOv11n, FEWheat-YOLO improved AP@50, AP_s, AP_m, AP_l, and AR by 0.53%, 0.7%, 0.7%, 0.4%, and 0.3%, respectively, while reducing parameters by 74%, computation by 15.9%, and model size by 69.2%. These results indicate that FEWheat-YOLO provides an effective balance between detection accuracy, counting performance, and model efficiency, offering strong potential for real-time agricultural applications on resource-limited platforms.

## 1. Introduction

Wheat is one of the most widely consumed cereal crops in the world. With the continuous growth of the global population [1], wheat consumption has increased steadily year by year [2], contributing approximately 35% and 74% of cereal-derived caloric intake in developing and developed countries, respectively [3,4]. Notably, wheat is not only a major source of carbohydrates and protein for human nutrition but also plays a critical role in the livestock sector. Approximately 20% of global wheat production is allocated to livestock feed [3,5], thereby indirectly supplying higher-quality protein to humans through energy conversion mechanisms. This “dual consumption structure” means that fluctuations in wheat production directly impact the security of staple food supplies and, through food chain transmission effects, exert cascading influences on meat production and the broader nutritional supply system. Therefore, accurately monitoring wheat growth and scientifically forecasting its yield are of great significance for ensuring food security and maintaining market stability [6,7].

In wheat breeding and yield assessment, three key indicators are widely recognized: the number of spikes per unit area, the number of grains per spike, and the thousand-kernel weight [8]. Among these, the number of spikes per unit area serves as an intuitive indicator reflecting wheat growth status and potential yield [9,10,11], and its accurate detection is particularly crucial for reliable wheat yield estimation.

In wheat spike detection tasks, traditional methods can generally be divided into two categories: manual inspection and classical image processing techniques. Manual methods are highly subjective and susceptible to human error, often resulting in misdetection and omission [12]. Furthermore, they are labor-intensive and not suitable for large-scale agricultural production [13]. As an alternative, traditional image processing techniques have been widely applied in early studies on spike detection and counting. For instance, JA Fernández Gallego et al. developed an automatic spike counting approach under natural lighting conditions using top-view RGB imagery [14]. Specifically, RGB images of wheat and barley canopies were captured in the field using a standard camera mounted on a tripod. The spike regions were then identified and counted through a combination of color segmentation, morphological operations, and contour analysis. While this method achieved promising counting accuracy under controlled conditions, it remains highly sensitive to factors such as camera angle, lighting variability, and background noise. Consequently, its generalization ability is limited in complex field environments with occlusion, weed interference, or morphological variation across cultivars, which constrains its applicability in large-scale real-world scenarios.

With the continuous advancement of deep learning, automatic wheat spike detection has gradually replaced traditional manual counting approaches, particularly with the widespread adoption of convolutional neural networks (CNNs) [15]. CNNs have been extensively applied in crop breeding and spike structure detection tasks [16,17,18]. Compared with traditional image-based methods, deep learning leverages computer vision to extract various image features such as morphology, color, and texture, enabling accurate crop detection and counting [19]. These techniques are widely used in breeding evaluation, growth monitoring, and yield estimation. CNN-based methods for detection and counting primarily fall into three categories: semantic segmentation, object detection, and density map regression [20].

Semantic segmentation methods rely on pixel-level annotations for training and prediction, enabling effective separation between crops and background, which makes them suitable for counting tasks [21,22,23,24]. However, such methods demand high annotation precision, require significant labor and time for data labeling, and typically place a higher burden on computational resources. Density map regression methods build a direct mapping between image features and target quantities and perform well in scenarios involving dense targets and heavy occlusion [25]. Nonetheless, these methods are sensitive to annotation quality and scene consistency, and they struggle with variations in target scale. Object detection methods generate candidate regions, classify and count objects, and are capable of detecting multiple targets in complex environments with high real-time performance. As a result, object detection has become one of the mainstream approaches in current wheat spike detection research [13].

Currently, wheat spike detection methods based on object detection are mainly categorized into two types: two-stage detectors and single-stage detectors [26]. Two-stage detectors typically employ a Region Proposal Network (RPN) to generate Regions of Interest (ROIs), followed by feature extraction and classification within these candidate regions. Representative models include R-CNN [27], SPP-net [22], Fast R-CNN [28], and Faster R-CNN [29]. These methods generally achieve high detection accuracy and are well-suited for fine-grained recognition in complex scenes. However, their multi-step processing pipelines result in relatively poor real-time performance.

In contrast, single-stage detectors dispense with the region-of-interest (ROI) proposal stage and perform bounding-box regression and object classification in a single pass over the entire image, which markedly improves inference speed. Representative models include You Only Look Once (YOLO) [30], Single Shot MultiBox Detector (SSD) [31], and RetinaNet [32]. Notably, RetinaNet introduces focal loss to mitigate extreme foreground–background class imbalance by down-weighting easy negatives, thereby improving detection in dense scenes and for small objects. Owing to their favorable accuracy–speed trade-off, single-stage detectors have become the mainstream approach for wheat spike detection and counting.

In practical applications, Li et al. [33] addressed the problem of wheat spike detection and cross-region counting in consecutive video frames. Based on the GWHD2021 dataset [34], an improved YOLOv7 [35] network was used for training. Combined with DeepSORT [36], a cross-line partitioned counting algorithm was designed, which effectively accomplished wheat spike detection and tracking. The improved YOLOv7 achieved a mean Average Precision at IoU = 0.5 (mAP@0.5) of 93.8%, an inference speed of 19.2 frames per second (FPS), and a model size of 57.7 MB. Here, mAP@0.5 refers to the mean Average Precision calculated at an IoU threshold of 0.5, commonly used in object detection tasks to measure model accuracy. Yang et al. [37] focused on the issue of wheat spike occlusion in complex backgrounds. They introduced RT-DETR as the base detector and optimized the network structure by considering the distribution characteristics of wheat spikes across multiple scales. This approach improved detection accuracy while reducing computational cost, thereby balancing speed and precision. The method achieved an average precision (AP) of 95.7, an FPS of 46.68, and a parameter count of 37 million. Guan et al. [9] proposed a hybrid architecture combining CNN and Transformer, in which the local features extracted by CNN were fed into the Transformer module to capture global context in the image. This design enhanced wheat spike detection performance under multi-scale field backgrounds. The method achieved an AP@50 of 93%, with a parameter count of 38.7 million and an FPS of 14.5. Although the above methods have achieved significant improvements in detection accuracy, their network structures are typically complex, with large parameter sizes and high computational demands, which results in relatively low FPS. Moreover, it is worth noting that the training and evaluation of these models are mostly conducted on high-end GPUs such as NVIDIA RTX 3090 or RTX A30, both equipped with 24 GB of VRAM, which greatly facilitates the training process. However, such high-performance GPUs are rarely available in real-world deployment scenarios, and the inference speed of these models is heavily influenced by the hardware performance. As a result, the actual inference speed in deployment may be lower than that reported in the literature. Hence, these models still face considerable challenges when applied in resource-constrained edge devices or real-world agricultural settings.

In recent years, with the growing demand for agricultural intelligence and edge deployment, some researchers have begun to explore lightweight models for wheat spike detection and counting. Chen et al. [38] trained their model on the GWHD2020 dataset and introduced BiFormer attention [39] along with other techniques to enhance the architecture. They employed transfer learning to validate the model on a UAV-captured wheat spike dataset, achieving a mAP of 94.01%, with only 1.34 million parameters and 3.9 GFLOPs, and a remarkable inference speed of 185 FPS. This approach significantly reduced the model’s complexity and computational cost while steadily improving detection accuracy, demonstrating strong potential for practical applications. Qing et al. [40] proposed another lightweight detection framework based on the YOLO-FastestV2 architecture. They integrated three attention mechanisms—Large Separable Kernel Attention (LSKA) [41], Multi-Scale Attention (EMA) [42], and Efficient Channel Attention (ECA) [43]—to enhance feature extraction in the backbone network. Additionally, they optimized the LightFPN structure using the SimConv [44] module. With only 0.39 GFLOPs and 0.24 million parameters, their model achieved an average precision (AP) of 81.52%, offering a cost-effective solution for agricultural automation.

Although the above studies have made notable progress in improving detection accuracy while reducing model complexity, a trade-off remains between performance and deployability in complex wheat field environments. High-accuracy models often incur substantial deployment costs, whereas models with lower resource demands tend to compromise precision. To balance detection accuracy and deployment efficiency, this study proposes a lightweight yet high-precision wheat spike detection framework. The proposed method optimizes the network architecture from multiple perspectives, including backbone design, attention mechanism integration, and feature fusion strategies, thereby achieving a synergistic improvement in both accuracy and computational efficiency. This framework demonstrates strong adaptability and scalability for real-world deployment. The specific methodology is detailed as follows:

(1) To enhance feature extraction capability, this paper proposes FEMANet (Faster-Enhanced Mixed Aggregation Network with Efficient Multi-scale Attention (EMA)), based on the Mixed Aggregation Network (MANet) [45]. This module is designed to strengthen feature representation while minimizing the number of parameters and overall model size, facilitating efficient deployment on resource-constrained devices.
(2) To address the issues of uneven scale distribution and loss of shallow details in wheat spike images, a Bidirectional Asymmetric Feature Aggregation Feature Pyramid Network (BiAFA-FPN) is introduced. This method aims to improve detection accuracy while further reducing the number of model parameters.
(3) To reduce redundant computation while preserving salient region information, this study incorporates the Adaptive Downsampling module (ADown), which improves computational efficiency while maintaining feature representation quality.
(4) In the design of the detection head, a Grouped Shared Convolution Detection Head(GSCDHead) is proposed. By integrating structural sharing and grouped convolution, GSCDHead effectively reduces computational cost while maintaining detection accuracy.

## 2. Materials and Methods

### 2.1. Hybrid Wheat Head Detection Dataset

To enhance the diversity of training samples and improve robustness to data distribution shifts, we integrate two datasets. The first is the public GWHD2021 dataset, and the second is a self-collected dataset. Together, these form a larger and higher-quality hybrid dataset. This dataset includes wheat spike images from multiple countries, captured under different conditions and involving various wheat cultivars. Additionally, it incorporates real-field samples collected under natural lighting conditions in the Xinjiang region of China. This further improves the dataset’s representativeness and practical value. For convenience, we refer to this hybrid dataset as the Hybrid Wheat Head Detection Dataset (HWHD).

#### 2.1.1. The GWHD2021 Public Dataset

**Figure 1:**
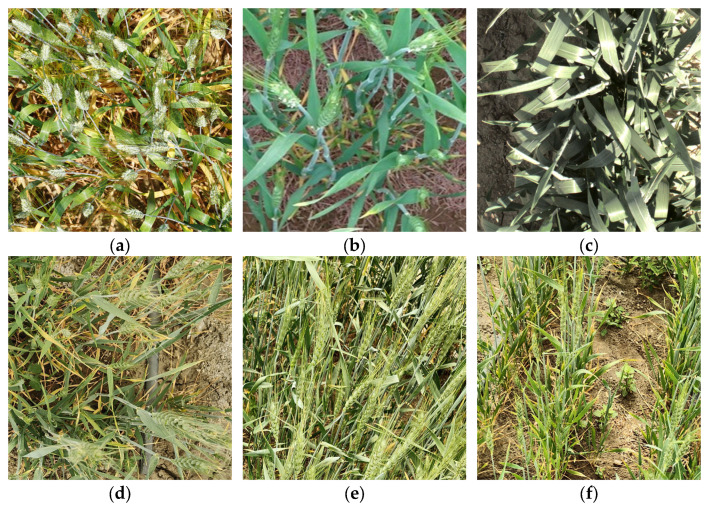
Representative wheat spike images from the two datasets used to construct the Hybrid Wheat Head Detection Dataset (HWHD). (**a**–**c**) Samples from the public dataset GWHD2021; (**d**–**f**) Samples from the custom dataset collected in Alar, Xinjiang, China (30 April–5 May 2024), during the heading stage of spring wheat.

GWHD is one of the most widely recognized wheat spike detection datasets in the world. Therefore, we selected the Global Wheat Head Detection 2021 (GWHD2021) as the public dataset for this study. GWHD2021 includes wheat spike images from multiple countries and regions, featuring different cultivars, and captured under varying conditions and with different cameras. This ensures the diversity and representativeness of the dataset. Representative samples from GWHD2021 are shown in Figure 1a–c.

The expanded GWHD2021 dataset consists of 6422 images contributed by 16 research institutions across 12 countries or regions, with a total of 275,187 annotated wheat heads. All images are standardized to a resolution of 1024 × 1024 pixels. The dataset covers multiple wheat cultivars, planting regions, camera angles, and acquisition devices, demonstrating strong diversity and representativeness.

#### 2.1.2. Collection and Preprocessing of the Custom Dataset

To enhance the generalization ability of the model, field data collection was conducted at the experimental site of the Meteorological Bureau in Alar City, Xinjiang, China (40.559° N, 81.227° E). The wheat was sown on 20 March 2024, using the cultivar Xinchun 40.

Images were captured from 30 April to 5 May 2024, during the daily time window of 11:00 to 16:00. At this stage, the wheat plants were in the heading phase, with most of them having developed green spikes. The weather conditions were favorable with abundant sunlight, and the selected time period corresponded to typical agricultural working hours, facilitating the potential applicability of the data in real-world agricultural scenarios.

**Figure 2:**
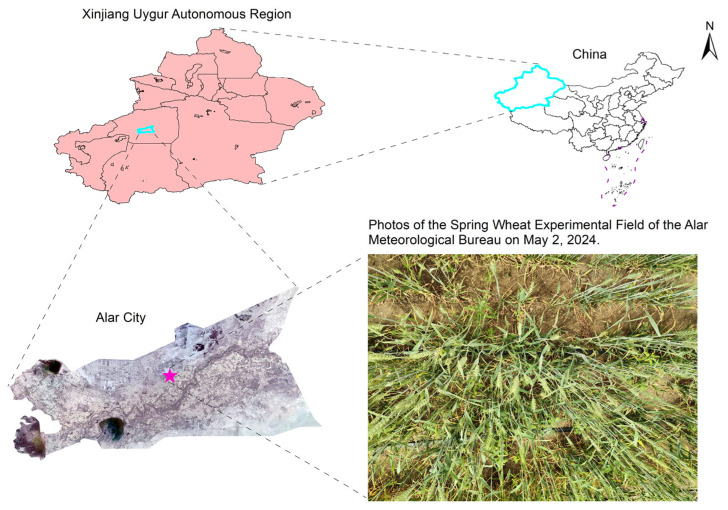
Spatial location of the wheat spike data collection area in Alar City, Xinjiang, China.

During the image acquisition process, a OnePlus Ace 2 smartphone (OnePlus, Shenzhen, Guangdong, China) was used to capture images of wheat spikes in a handheld manner from multiple viewing angles, including vertically overhead as well as oblique angles of approximately 45° and 60°. The smartphone’s camera, equipped with a Sony IMX890 50 MP sensor (Sony, Tokyo, Japan), automatically applied filters and white balance adjustments to optimize image quality. Images were captured in portrait mode with the default camera settings, ensuring proper focus, exposure, and continuous autofocus (AF). These settings were automatically adjusted by the camera’s software to ensure optimal image quality under various conditions. The camera-to-target distance was maintained within a range of 40–60 cm to ensure high image clarity and adequate field-of-view coverage. The images were saved in JPEG format, each with a resolution of 3072 × 4096 pixels. A total of 400 original images were collected using this approach (Figure 2).

An image tiling technique was applied to optimize the dataset structure and improve its suitability for training and testing deep learning models. Specifically, each original image was cropped into multiple sub-images of 1024 × 1024 pixels using the Python Imaging Library (PIL). To preserve as much useful information as possible and reduce the risk of truncating wheat spikes at the edges, a 10% overlap was applied. For edge regions that were smaller than 1024 × 1024 pixels after cropping, white padding was used to complete the image to the standard size, ensuring structural consistency across the dataset.

Through this preprocessing pipeline, approximately 2300 sub-images were initially generated. These images were then manually reviewed by the research team, and blurred, overexposed or underexposed, poorly focused, and otherwise unrepresentative samples were removed. After rigorous quality control, a total of 2200 high-quality images were retained as the final custom dataset. Image annotation was carried out using the Labelme tool, and the entire process was completed by a single graduate student with relevant expertise to ensure consistency and accuracy in the annotations.

Finally, the public dataset GWHD2021 and the custom dataset were merged to construct the Hybrid Wheat Head Detection Dataset (HWHD), which was subsequently split into training, validation, and test sets at a ratio of 7:2:1. The number of images in each subset is detailed in Table 1.

**Figure 3:**
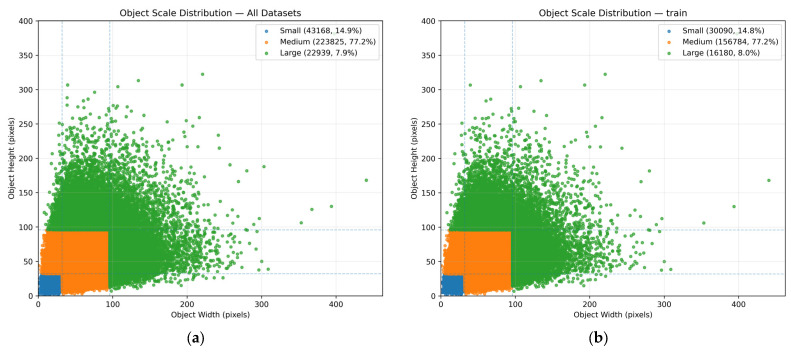

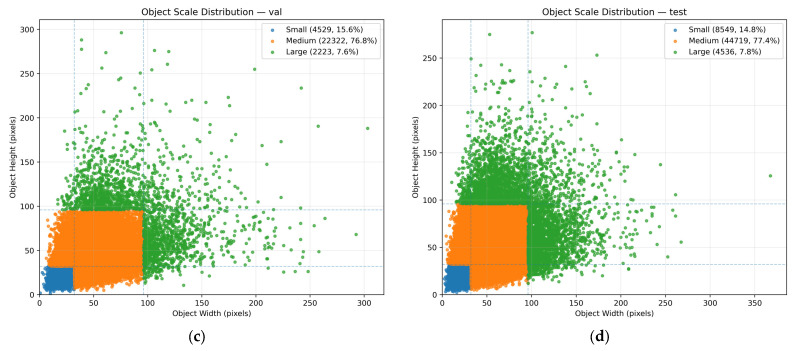
Object scale distribution of wheat spikes in the HWHD dataset, categorized by COCO size thresholds. (**a**) Distribution for all datasets. (**b**) Distribution for the training set. (**c**) Distribution for the validation set. (**d**) Distribution for the test set.

A statistical analysis of object scales in the Hybrid Wheat Head Detection Dataset (HWHD) was conducted, as illustrated in Figure 3. We define the scale of a wheat spike using the larger of its width or height. Following the COCO evaluation criteria [46], objects smaller than 32 × 32 pixels are categorized as small-scale, those between 32 × 32 and 96 × 96 pixels as medium-scale, and those larger than 96 × 96 pixels as large-scale. Note that this analysis is based on an input image size of 640 × 640 pixels.

As shown in the figure, 14.9% of wheat spikes are small-scale, 77.2% are medium-scale, and 7.9% are large-scale. The combined proportion of small and medium objects reaches 92.1%, indicating that wheat spike detection involves a significant challenge due to the dominance of small targets.

### 2.2. FEWheat-YOLO Network Architecture

The YOLO (You Only Look Once) series has become a leading single-stage object detection framework in computer vision. YOLO’s end-to-end prediction, fast detection speed, and simple architecture have led to its widespread use in real-time applications. Over successive versions, YOLO has balanced accuracy and efficiency, from the multi-scale prediction in YOLOv3 [47] to the lightweight design in YOLOv5 [48] and the architectural refinements in YOLOv8 [49]. YOLO11 [50] builds on this evolution, enhancing multi-scale target perception and feature expressiveness. It comprises three components: Backbone, Neck, and Head. The input image undergoes Mosaic augmentation, then passes through the Backbone for multi-scale feature extraction, combining depthwise separable convolutions with an improved C3k2 module. The Neck uses FPN and PAN to facilitate information flow across scales, while the C2PSA module provides channel-spatial attention. The Head applies parallel detection heads across three scales (P3/8, P4/16, P5/32) to detect small, medium, and large objects, improving localization accuracy while maintaining efficient inference.

**Figure 4:**
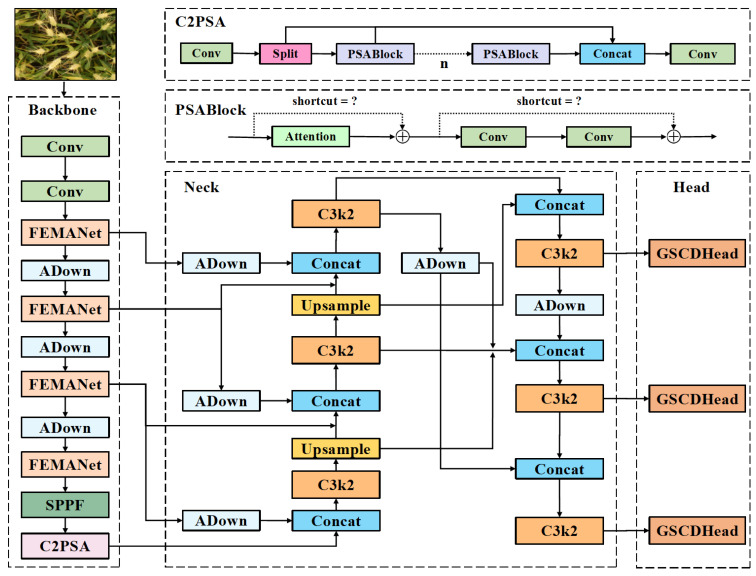
Comprehensive architecture of the proposed FEWheat-YOLO model.

In this study, YOLOv11n is selected as the baseline model because of its minimal parameter size and computational cost. These features make it well-suited for deployment on mobile devices and edge applications. Based on this foundation, we propose FEWheat-YOLO, a lightweight and performance-enhanced model tailored for wheat spike detection. The overall architecture of the proposed model is illustrated in Figure 4. Aimed at addressing the challenges posed by dense object distribution, small object scale, and morphological diversity in wheat spike detection, FEWheat-YOLO integrates four key improvement modules. First, a mixed aggregation, multi-scale adaptive feature-enhancement module (FEMANet) is introduced to improve the backbone’s semantic understanding of small- and medium-sized objects, enabling scale-aware feature representation. Second, a lightweight bidirectional feature aggregation structure (BiAFA-FPN) is designed to reconstruct the topological connections in the FPN, thereby enhancing the efficiency and complementarity of feature fusion between deep and shallow layers. Third, a content-aware adaptive downsampling operator (ADown) is employed to dynamically preserve important structural features of wheat spikes while reducing computational complexity. Fourth, a Grouped Shared Convolution Detection Head(GSCDHead) is developed to significantly reduce model size and inference overhead through parameter sharing and task decoupling mechanisms, without compromising detection accuracy. Together, these modules form the core architecture of FEWheat-YOLO, enabling efficient and accurate detection of multi-scale wheat spike targets while maintaining lightweight characteristics and deployment-friendliness.

#### 2.2.1. FEMANet

**Figure 5:**
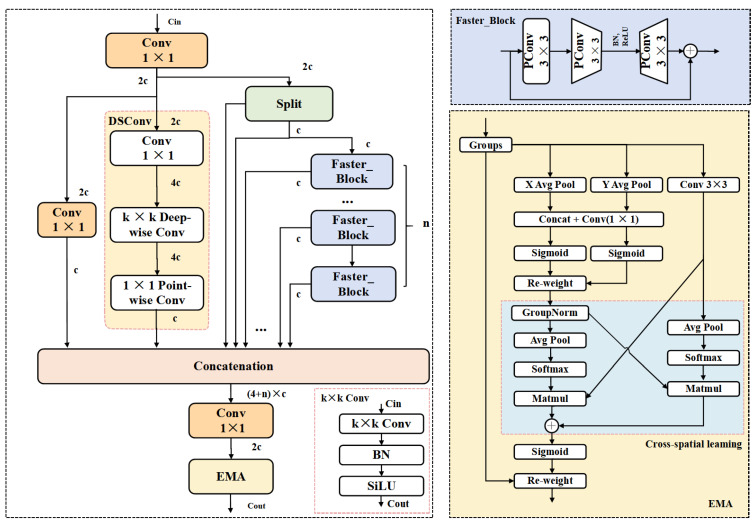
The architecture of FEMANet.

In natural field environments, wheat spikes often exhibit highly dense distributions, with significant overlap and occlusion. The boundaries between individual targets tend to be blurred. As a result, detection models struggle to accurately distinguish each wheat spike. Moreover, the morphology and scale of wheat spikes vary considerably depending on the growth stage, shooting angle, and lighting conditions, further increasing the detection complexity. The field background is also cluttered, with various interfering elements such as weeds, soil, and leaves, which can easily lead to false positives and missed detections. In YOLOv11, the Backbone employs the C3k2 module as its primary feature extraction unit. However, due to its fixed convolutional kernel combinations and limited spatial receptive field, this module struggles to effectively distinguish densely packed and occluded wheat spike targets. To address these limitations, this study draws inspiration from the EMA mechanism [42], MANet [45], and FasterNet [51], and proposes an improved backbone feature extraction module—Faster-Enhanced Mixed Aggregation Network with EMA (referred to as FEMANet). FEMANet is designed to replace the original C3k2 module and enhance the model’s detection capability under challenging conditions such as densely distributed wheat spikes, heavy occlusion, large scale variations, and complex background interference. The network architecture of FEMANet is illustrated in Figure 5.

As shown in Figure 5, FEMANet consists of three types of feature extraction paths: (1) a channel compression path using 1 × 1 convolutions; (2) a spatial modeling path based on Depthwise Separable Convolution [47]; and (3) a multi-layer residual aggregation path composed of repeated Faster_Blocks. The output of the module is obtained by fusing and recalibrating the semantic features from these three paths, and the computation process is defined in Equation (1).

(1) $$\left\{\begin{array}{l} X_{mid}=Conv_{1}(X_{in})\\ X_{1}=Conv_{2}(X_{mid})\\ X_{2}=DSConv(Conv_{3}(X_{mid}))\\ X_{3},X_{4}=Split(X_{mid})\\ X_{5}=Faster\_Block_{1}(X_{4})+X_{4}\\ X_{6}=Faster\_Block_{2}(X_{5})+X_{5}\\ \ldots \\ X_{4+n}=Faster\_Block_{n}(X_{3+n})+X_{3+n} \end{array}\right\}$$

Among them, the number of channels in $X_{mid}$ is 2c, which is generated from the input feature map $X_{in}$ through $Conv_{1}$. Based on $X_{mid}$, different feature maps are obtained through specific operations: the number of channels in $X_{1}$ is c, obtained from $X_{mid}$ using $Conv_{2}$; the number of channels in $X_{2}$ is 2c, obtained from Xmid using Depthwise Separable Convolution (DSConv); and the number of channels in $X_{3}$ and $X_{4}$ is c, obtained from $X_{mid}$ by a Split operation. Subsequently, a series of residual feature maps $X_{5}$, $X_{5}$, …, $X_{6}$ are generated by stacking repeated Faster_Blocks, and each of these residual feature maps also has c channels. Finally, all semantic features $X_{1},X_{2},X_{3},\ldots ,X_{4+n}$ are fused and compressed Via a concatenation operation to form the output of the module.

For the Faster_Block, let the input feature be $X\in {\mathbb{R}}^{c\times H\times W}$, and the output be Y. The main computation process of the Faster_Block is as follows:

(2) Z=P(X)

(3) $$U=W_{2}*\sigma (W_{1}*Z)$$

(4) Y=X+DropPath(U)

Specifically, P(⋅) denotes spatial convolution operations applied to partial channels, ∗ represents convolution operations, and σ(⋅) is the activation function. $W_{1}$ and $W_{2}$ are two 1 × 1 convolution kernels. Through residual connections, the input is fused with the nonlinear transformation output to enable stable and efficient feature learning.

To further enhance the model’s discriminative ability for target regions and improve detection accuracy under complex backgrounds, we introduce the Enhanced Mask Attention (EMA) mechanism. This module leverages global average pooling and max pooling of the input feature maps to extract various types of contextual information, generating a spatial attention weight map to explicitly enhance key regions.

Specifically, given an input feature map $X\in {\mathbb{R}}^{C\times H\times W}$, the EMA module first performs global average pooling and max pooling across the spatial dimensions to obtain two channel-wise attention cues, formulated as:

(5) $$F_{avg}=AvgPool(X),\;F_{\max}=MaxPool(X)$$

Then, the two cues are concatenated along the channel dimension and fed into a convolutional layer to generate the spatial attention map:

(6) $$W=\sigma \left(Conv\left(\left[F_{avg},F_{\max}\right]\right)\right)$$

Here, [⋅] denotes the concatenation operation, and $W\in {\mathbb{R}}^{1\times H\times W}$ represents the spatial attention weights for each spatial position. Finally, the attention weights are applied to the input feature map Via element-wise multiplication to explicitly enhance the target regions:

(7) $$X_{out}=W⊙X$$

Here, ⊙ denotes the element-wise multiplication. EMA effectively enhances the model’s perception of wheat spike regions while suppressing interference from non-target areas, thereby improving the discriminability and stability of the feature maps.

Finally, the target-aware features are refined through a 1 × 1 convolution and the EMA module, resulting in the output feature $X_{out}$ with 2c channels. The computation is formulated as:

(8) $$X_{out}=EMA\left(Conv\left(X_{1}||X_{2}||\ldots ||X_{4+n}\right)\right)$$

where || denotes the concatenation operation along the channel dimension.

#### 2.2.2. BiAFA-FPN

Due to the relatively small spatial extent of wheat spikes in field images, their features and positional information are prone to gradual loss as the network depth increases, ultimately affecting detection accuracy. In the original YOLOv11 architecture, PANet is adopted as the neck feature fusion module. However, this design still suffers from a high rate of false positives and missed detections for small-scale wheat spikes, failing to meet the requirements for fine-grained recognition.

**Figure 6:**
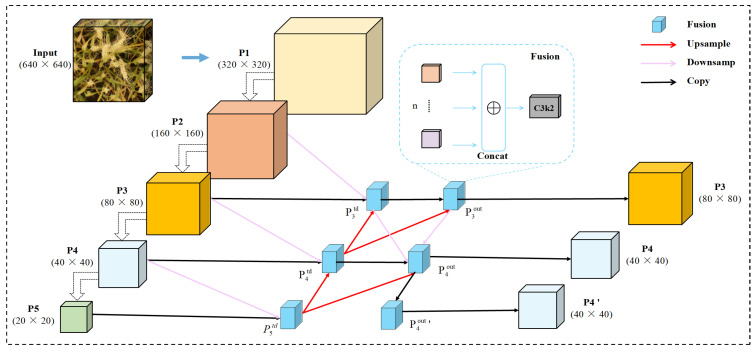
Architecture of the proposed BiAFA-FPN module. Multi-scale features are concatenated (Concat) and processed Via a C3k2 block to enhance feature fusion. Red arrows indicate upsampling operations, purple arrows represent downsampling operations, and black arrows denote copy connections. The inset illustrates the internal structure of the fusion node using the C3k2 module.

To address these limitations, we propose an improved feature fusion structure named BiAFA-FPN (Bidirectional Asymmetric Feature Aggregation Feature Pyramid Network), specifically designed for the dense distribution, small scale, and weak edge characteristics of wheat spikes. As illustrated in Figure 6, red arrows indicate upsampling operations, while purple arrows represent downsampling operations implemented Via convolution. In the feature fusion stage, semantic information is aggregated using feature-level concatenation. We have evaluated several fusion strategies in comparative experiments, and the detailed performance will be discussed in the experimental section. The design of BiAFA-FPN is inspired by MAFPN [52], BiFPN [53], and FBRT-YOLO [54], and further extended and optimized for lightweight deployment. The resulting structure outputs features at two different scales: the P3 layer is responsible for detecting small targets and is output once; the P4 layer is used for medium targets and is output twice; the P5 layer is omitted due to its excessively large receptive field, which contributes little to small object detection while increasing computational redundancy.

The overall BiAFA-FPN consists of two complementary paths: a top-down and a bottom-up pathway:

In the top-down path, deep semantic features are fused with high-resolution features at the same level and shallow features from the backbone, thereby enhancing the network’s sensitivity to small and medium-sized objects;

In the bottom-up path, the fusion sources include shallow high-resolution features, shallow low-resolution features, same-level features, and features from the previous fusion stage. As a result, the P4 layer integrates semantic information from four different levels, while the P3 layer aggregates features from two levels.

As shown in Figure 6, the proposed BiAFA-FPN consists of two main pathways: a top-down path and a bottom-up path. In the top-down pathway, deep semantic features are fused with high-resolution features at the same level and shallow features from the backbone network. This strategy effectively preserves the spatial location and contextual semantics of small and medium-sized targets, thereby enhancing the network’s perception capability for fine-grained objects.

The feature fusion process can be formulated as:

(9) $$P_{5}^{td}=C\left(P_{5}^{in}+R\left(P_{4}^{in}\right)\right)$$

(10) $$P_{4}^{td}=C\left(P_{4}^{in}+R\left(P_{3}^{in}\right)+R\left(P_{5}^{in}\right)\right)$$

(11) $$P_{3}^{td}=C\left(P_{3}^{in}+R\left(P_{2}^{in}\right)+R\left(P_{4}^{in}\right)\right)$$

Among them, $P_{i}^{td}$ denotes the feature map between layers $C_{i}$ and $P_{i}^{in}$, where i represents the current layer. $P_{i}^{in}$ refers to the input of layer $C_{i}$, and R(X) indicates the downsampling operation. C(X) represents the convolution operation after concatenation and fusion.

To improve detection efficiency, we did not adopt the P2 detection head. Therefore, in actual deployment, the P3 layer only fuses information from two different levels rather than all four, thus avoiding a significant increase in computational cost and preventing error accumulation.

In the bottom-up pathway, low-resolution features from shallow layers are upsampled and fused with high-level semantic information from previous layers. The final outputs of P3 and P4 layers after the second fusion are as follows:

(12) $$P_{3}^{out}=C\left(P_{3}^{td}+R\left(P_{4}^{td}\right)\right)$$

(13) $$P_{4}^{out}=C\left(P_{4}^{td}+R\left(P_{5}^{td}\right)+R\left(P_{3}^{td}\right)+P_{3}^{out}\right)$$

(14) $$P_{4}^{{\mathrm{out}}^{\prime }}=C\left(R\left(P_{3}^{td}\right)+P_{4}^{out}\right)$$

Here, $P_{3}^{out}$ is the output of $P_{3}$, $P_{4}^{out}$ and $P_{4}^{{\mathrm{out}}^{\prime }}$ correspond to the two output branches of the P4 layer. To ensure detection accuracy, this dual-branch output strategy significantly enhances the robustness of medium-object detection while aligning with the lightweight design requirements.

#### 2.2.3. ADown

In natural environments, wheat spikes often blend into the background, and their texture features tend to be similar to surrounding elements. For instance, during the early or mid-heading stages, wheat spikes and leaves usually appear in similar green tones, resulting in blurred object boundaries and significantly increased detection difficulty. Traditional convolution-based downsampling methods (e.g., Conv) often lead to the loss of fine-grained features, especially in lightweight models with limited parameters.

**Figure 7:**
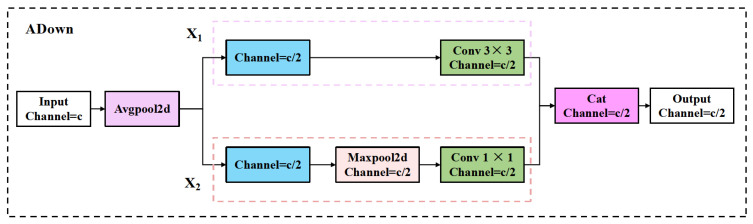
The architecture of the proposed ADown (Adaptive Downsampling) module.

To address this issue, we introduce a compact and responsive adaptive downsampling module (ADown) [55], which is designed to preserve critical regional features during the downsampling process and enhance the model’s perceptual ability for wheat spike targets. As illustrated in Figure 7 ADown, the ADown module consists of two parallel branches, which fuse different types of spatial information to improve the overall representational capacity.

Specifically, the input features are first subjected to average pooling for initial spatial downsampling:

Then, the pooled features are split into two groups along the channel dimension:

(15) $$X_{pool}=P_{avg}\left(X;k=2,s=1\right)$$

(16) $$X_{1},X_{2}=S\left(X_{pool};\dim=1\right)$$

The first group $X_{1}$ is processed by a 3 × 3 convolution to extract low-level features:

(17) $$Y_{1}=C_{3\times 3}(X_{1};s=2),\;Y_{1}\in {\mathbb{R}}^{\frac{H}{2}\times \frac{W}{2}\times C^{\prime }}$$

The second group $X_{2}$ is first enhanced using max pooling to highlight salient regions, followed by a 1 × 1 convolution for channel integration:

(18) $$Y_{2}=C_{1\times 1}(P_{\max}(X_{2};k=3,s=2)),\;Y_{2}\in {\mathbb{R}}^{\frac{H}{2}\times \frac{W}{2}\times C^{\prime }}$$

Finally, the two branches are concatenated along the channel dimension to obtain a fused multi-scale spatial representation:

(19) $$Y=F_{cat}(Y_{1},Y_{2}),\;Y\in {\mathbb{R}}^{\frac{H}{2}\times \frac{W}{2}\times 2C^{\prime }}$$

This structure is designed with lightweight efficiency in mind, aiming to retain critical spatial information of wheat spike targets while minimizing computational overhead, making it suitable for deployment in resource-constrained environments such as mobile or edge devices.

#### 2.2.4. GSCDHead

In the design of YOLOv11, the detection head is responsible for converting multi-scale feature maps generated by the backbone and feature pyramid network into final bounding box regression and category prediction outputs. The original design adopts separate convolutional branches for each detection scale, which allows for flexible configuration and high accuracy in large-object detection. However, this structure tends to introduce parameter redundancy and information duplication in dense scenes or small-object scenarios, thus limiting the deployment efficiency of YOLOv11 in lightweight models and fine-grained detection tasks.

To address the issues of parameter redundancy and computational inefficiency in the YOLOv11 detection head, this paper proposes a Grouped Shared Convolution Detection Head(GSCDHead). Unlike the scale-specific independent structure in YOLOv11,GSCDHead introduces a shared convolutional path and incorporates Group Normalization (GroupNorm) [56] to normalize the extracted features, thereby reducing parameter count and resource consumption while improving computational efficiency and feature modeling stability. Additionally, Group Convolution [57] is adopted to further compress the computational complexity.

**Figure 8:**
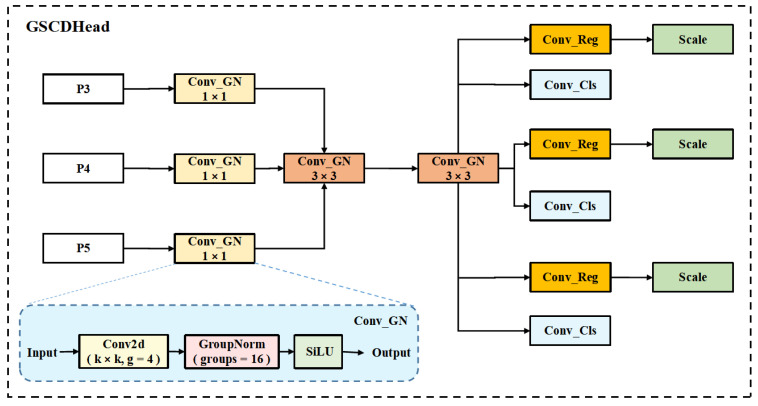
The proposed GSCDHead module structure, which illustrates the shared convolution backbone and its internal components. Here, k × k denotes the kernel size, and g represents the number of groups in grouped convolution.

Each detection branch is equipped with an independent Scale module to modulate output response intensity, thereby enhancing scale adaptability. This detection head design balances lightweight architecture and representational capability, enabling efficient sharing and fusion of multi-scale semantic information while maintaining the advantage of a reduced parameter count. The architecture of the GSCDHead is illustrated in Figure 8.

First, feature map transformation is performed. Let the input feature map set be denoted as ${\left\{x_{i}\in {\mathbb{R}}^{B\times C_{i}\times H_{i}\times W_{i}}\right\}}_{i=1}^{N}$, where N is the number of detection layers. $x_{i}$ is first processed through an independent convolutional block:

(20) $$x_{conv}[i]=Conv_{GN}(x_{i},hidc,3)$$

The output dimensions are $B\times hidc\times H_{i}\times W_{i}$. Where B denotes the batch size, hidc is the number of output channels, and, $H_{i}$, $W_{i}$ are the height and width of the feature map in the i-th layer. The computation of $Conv_{GN}$ is defined as:

(21) $$x_{b,g}^{\prime }=\frac{x_{b,g}-\mu_{g}}{\sqrt{\sigma_{g}^{2}+\int }}\cdot \gamma_{g}+\beta_{g}$$

$\mu_{g},\sigma_{g}$ denote the mean and standard deviation of each group channel; $\gamma_{g},\beta_{g}$ are the learnable scaling and shifting parameters; g is the group index, and the number of groups is equal to the number of channels.

Then, the features from each layer are passed through shared depthwise separable convolutions:

(22) $$x_{share}[i]=Conv_{GN}(Conv_{GN}(x_{conv}[i],hidc,3,g=hidc),hidc,1)$$

The first convolution operation adopts group convolution (i.e., g=hidc), which divides the input channels into g=hidc groups, each convolved independently to effectively reduce parameter count. The second convolution is a 1 × 1 convolution used for channel fusion and adjustment.

Next, bounding box regression and classification prediction are performed. For bounding box regression:

(23) $$bbox\_pred[i]=Scale\left(cv2(x_{share}[i])\right)$$

Here, the output dimension of cv2 is $4\times reg_{-}\max$. After appropriate scaling and reshaping, the DFL decoding is applied as:

(24) $$coord=\sum_{d=0}^{reg\_\max-1}Soft\max\left(bbox\_pred{\left[i\right]}_{d}\right)\cdot d$$

where $reg_{-}\max$ represents the number of discrete bins and d denotes the index of each bin.

For classification prediction:

(25) $$cls\_prob[i]=\sigma \left(cv3(x_{share}[i])\right)$$

where σ denotes the Sigmoid activation function.

During training, the predictions from each detection head are returned independently.

During inference, the outputs from all detection heads are concatenated and subsequently decoded to obtain the final detection results.

(26) $$x_{cat}=Concat\left(reshape(bbox\_pred,\;cls\_prob)\right)$$

The final coordinates are decoded using anchor boxes and stride.

### 2.3. Experimental Setup

To ensure the fairness of the training and validation processes, all experiments in this study were conducted under a consistent software and hardware environment.

Hardware Configuration:

The experimental platform was equipped with an AMD Ryzen 5 5500 processor (AMD, Santa Clara, CA, USA), 48 GB of system memory, an NVIDIA GeForce RTX 3070 Ti graphics card (NVIDIA, Santa Clara, CA, USA)(8 GB VRAM), and a 1 TB solid-state drive, providing sufficient computational power and storage support, effectively preventing interruptions or instability due to hardware performance limitations.

Software Environment:

The operating system used was Windows 10, with a Windows Subsystem for Linux (WSL) environment established to install dependencies like PyTorch 2.1.1 and NumPy 1.24.4. The WSL environment was based on Ubuntu 20.04 LTS, suitable for model building, training, and evaluation. The Python environment was configured with Python 3.11.5, PyTorch 2.1.1, and CUDA 11.8, which improved training efficiency while minimizing dependency-related issues.

Considering the characteristics of the FEWheat-YOLO model and the stability requirements of the experiments, a unified set of hyperparameters was used across all experimental runs, as shown in Table 2:

In addition to the primary experimental setup, the model was deployed on a Raspberry Pi 4 Model B (Raspberry Pi Foundation, Cambridge, UK)(4 GB RAM) for edge device testing, running a 32-bit operating system, to assess the model’s real-time inference capability in resource-constrained environments, simulating potential deployment scenarios in agricultural automation. The Python environment on the Raspberry Pi was fully synchronized with that on the PC, ensuring consistency across platforms. To evaluate the performance of different methods on the Raspberry Pi, we converted the FEWheat-YOLO model into ONNX, OpenVINO, Paddle, and NCNN formats, and installed the corresponding Python packages on the Raspberry Pi to support the execution of these formats.

### 2.4. Evaluation Indicators

To comprehensively evaluate the performance of the model, this study analyzes both accuracy and inference efficiency. The model complexity is measured by the number of floating-point operations (FLOPs) and the number of parameters (Params), while the inference speed is evaluated using Frames Per Second (FPS).

In object detection tasks, YOLO-series models commonly utilize a confusion matrix to assess classification performance. The confusion matrix includes four basic indicators: True Positive (TP), False Positive (FP), True Negative (TN), and False Negative (FN). Based on these values, Precision and Recall can be further calculated, as defined by Equations (27) and (28), respectively:

(27) $$P_{r}=\frac{TP}{TP+FP}$$

(28) $$R_{e}=\frac{TP}{TP+FN}$$

Precision (P) measures the accuracy of the model’s detection results, indicating the proportion of correctly detected wheat spikes among all positive predictions. Recall (R) reflects the model’s ability to identify actual positive samples, representing the proportion of correctly detected wheat spikes among all ground-truth wheat spikes.

Since there is a trade-off between precision and recall, a single metric cannot comprehensively assess model performance. Therefore, this study adopts Average Precision (AP) as the core evaluation metric. AP measures detection performance across various recall levels within the range of 0 to 1, and its computation is defined as follows:

(29) AP=∫01Pr(Re)d Re

Specifically, we use AP@50 to denote the average precision when the Intersection over Union (IoU) threshold is set to 0.5. AP_s represents the detection accuracy for small-scale wheat spikes, AP_m for medium-scale wheat spikes, and AP_l for large-scale wheat spikes.

These metrics reflect the model’s generalization ability and detection accuracy across multiple target scales from different perspectives, and together they form the core criteria for evaluating the performance of the FEWheat-YOLO model in this study.

In addition, we evaluate the model’s performance in wheat spike counting tasks using the coefficient of determination (R^2^), Mean Absolute Error (MAE), and Root Mean Square Error (RMSE). R^2^ measures the degree of fit between predicted values and ground truth, ranging from 0 to 1. A value closer to 1 indicates better model fitting and more accurate predictions. MAE reflects the average magnitude of errors between predicted and actual values. RMSE indicates the variability of prediction errors and reflects the stability of the prediction results.

The corresponding calculation formulas are presented in Equations (30)–(32).

(30) $$R^{2}=1-\frac{SS_{res}}{SS_{tot}}=1-\frac{\sum_{i=1}^{n}{\left(y_{i}-{\hat{y}}_{i}\right)}^{2}}{\sum_{i=1}^{n}{\left(y_{i}-\bar{y}\right)}^{2}}$$

(31) $$MAE=\frac{1}{n}\sum_{i=1}^{n}|y_{i}-{\hat{y}}_{i}|$$

(32) $$RMSE=\sqrt{\frac{1}{n}\sum_{i=1}^{n}{\left(y_{i}-{\hat{y}}_{i}\right)}^{2}}$$

where n denotes the number of images, i represents the index of the i-th image, $y_{i}$ is the actual number of wheat spikes in the i-th image, ${\hat{y}}_{i}$ is the predicted count by the model, and $\bar{y}$ is the average number of wheat spikes across all images.

In summary, lower values of MAE and RMSE indicate better performance, as they reflect smaller error and less variability between predictions and actual values. On the other hand, a higher R^2^ value implies stronger goodness of fit, showing that the model better captures the distribution of real-world data.

### 2.5. Metric Measurement Methods

All results in this study are evaluated on the HWHD test set, with the validation set used solely for model selection. Detection performance is assessed using COCO-style metrics, including Average Precision (AP), AP@50, scale-specific AP values (AP_s, AP_m, AP_l), and Average Recall (AR). All metrics and hyperparameters are computed under the data split (7:2:1 for training/validation/testing) and input size (640 × 640) conditions described in Section 2.3 and Section 2.4, with Mosaic augmentation applied.

To evaluate the impact of different model architectures on detection performance, we designed several comparative experiments, focusing on the effects of Backbone selection, FPN structure, feature fusion methods, and downsampling strategies. Specifically, starting from YOLOv11, we compared different Backbones to select the optimal performing backbone. Building upon this, we compared various FPN structures and further evaluated different feature fusion strategies within the FPN, ultimately selecting the best fusion method. Based on these selections, we also compared different downsampling strategies (such as ADown) and conducted experiments under the same dataset split and training settings to ensure fairness in the results.

The model’s scale and complexity, including parameter count (Parameters), Floating Point Operations (GFLOPs), and model size, are automatically computed using official APIs or Python scripts. Model size is calculated using FP16 weights (2 bytes per parameter); for models that are by default saved in FP32 precision (4 bytes per parameter), the reported values correspond to their FP16-equivalent size to ensure consistency and fairness in comparison. Inference speed (FPS) is measured on the same hardware platform used for training, with a batch size set to 1, using FP16 precision, single-threaded data loading, and an initial warm-up of 50 images. The average inference time for 500 test images is recorded.

## 3. Results

**Figure 9:**
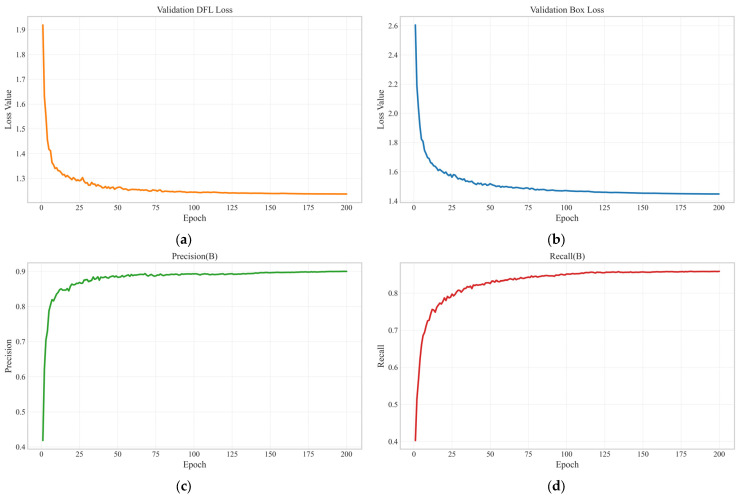
Training metrics of the FEWheat-YOLO model on the HWHD validation set: (**a**) validation distribution focal loss (DFL); (**b**) validation box loss; (**c**) precision; (**d**) recall.

During training, the Mosaic data augmentation strategy was enabled throughout the entire training process to enhance the model’s generalization capability under complex scenarios. Figure 9 presents the validation performance of the FEWheat-YOLO model on the HWHD dataset, showing the evolution of the validation distribution focal loss (DFL), validation box loss, precision, and recall, which reflects the model’s convergence and performance improvement throughout training.

### 3.1. Comparison with State-of-the-Art Methods

To comprehensively evaluate the performance of the proposed FEWheat-YOLO model in detecting wheat spikes under complex natural conditions, we conducted comparative experiments on the HWHD dataset against mainstream object detection models, including Faster R-CNN [29], SSDLite-MobileNetV2 [31,58], RT-DETR-R18 [59], as well as lightweight YOLO variants ranging from YOLOv5-n, YOLOv6-n, YOLOv7-Tiny, YOLOv8-n, YOLOv9-t, YOLOv10-n [60], YOLOv12-n [61], to YOLOv13-n [62]. All experiments were performed under a unified hardware and software environment with consistent hyperparameter settings to ensure fair comparisons.

The results show that FEWheat-YOLO achieves outstanding performance with minimal computational cost. It outperformed all other models with an average precision (AP) of 51.11%, an AP@50 of 89.8%, and medium-object detection accuracy (AP_m) of 50.5%. The average recall (AR) reaches 58.1%, which exceeds most comparison models and is close to the highest AR (58.3%) achieved by YOLOv10-n. For small object detection, FEWheat-YOLO and RT-DETR-R18 both achieve the highest AP_s of 18.1%, representing an 8.6 percentage point improvement over Faster R-CNN, demonstrating the model’s strong capability in detecting dense and small-scale wheat spikes.

Regarding model complexity and inference efficiency, FEWheat-YOLO only uses 0.673 M parameters, 5.3 GFLOPs, and a model file size of 1.6 MB, achieving an inference speed of 54 FPS. Although slightly slower than YOLOv5-n, YOLOv6-n, and YOLOv7-Tiny, the proposed model outperforms YOLOv9-t, YOLOv13-n, and Faster R-CNN, and performs comparably to YOLOv12-n, ranking at a moderate level among all models. Detailed results are provided in Table 3.

**Figure 10:**
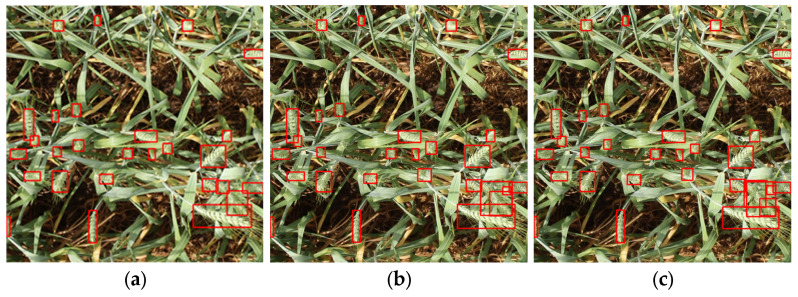

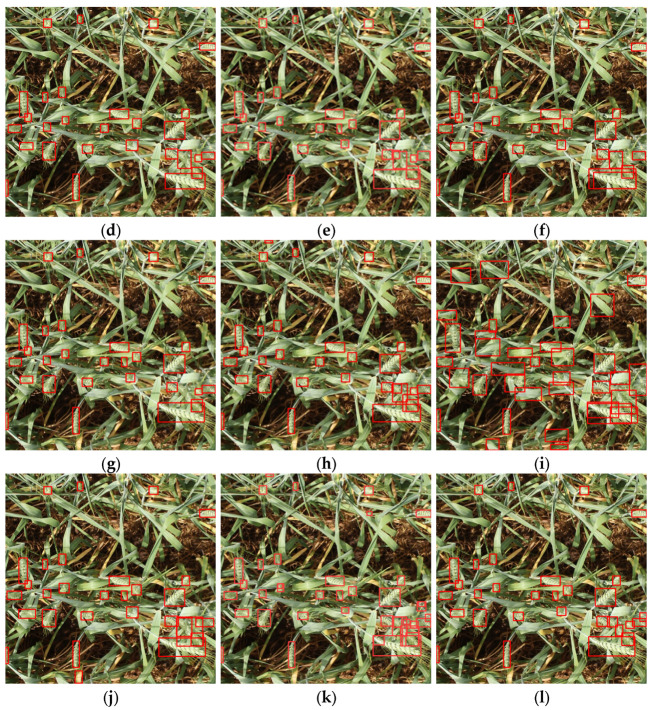
Qualitative comparison of wheat spike detection results using different models. (**a**) YOLOv5-n; (**b**) YOLOv6-n; (**c**) YOLOv7-Tiny; (**d**) YOLOv8-n; (**e**) YOLOv9-t; (**f**) YOLOv10-n; (**g**) YOLOv12-n; (**h**) YOLOv13-n; (**i**) Faster R-CNN; (**j**) SSDLite-MobileNetV2; (**k**) RT-DETR-R18; (**l**) Ours. The red boxes indicate the wheat spikes predicted by the models.

To further demonstrate the qualitative effectiveness of different models in wheat spike detection, we present visual comparisons between FEWheat-YOLO and several representative detectors, including YOLOv5-n, YOLOv6-n, YOLOv7-Tiny, YOLOv8-n, YOLOv9-t, YOLOv10-n, YOLOv12-n, YOLOv13-n, Faster R-CNN, and RT-DETR-R18, as illustrated in Figure 10.

Under natural field conditions with complex backgrounds and low contrast between wheat spikes and surrounding foliage, many models exhibit varying degrees of false positives and missed detections, especially in cases of occlusion and blurred spike boundaries. In contrast, FEWheat-YOLO achieves more precise localization, with fewer detection errors and better consistency across diverse scenarios. These visual results confirm the model’s improved robustness in challenging field environments.

### 3.2. Wheat Spike Counting Performance

To further evaluate the practical effectiveness of the proposed model in wheat spike counting, we conducted a comparative experiment against a baseline method. The evaluation employed three standard metrics: mean absolute error (MAE), root mean square error (RMSE), and the coefficient of determination (R^2^).

As shown in Table 4, FEWheat-YOLO achieved the best performance, with an MAE of 3.46, RMSE of 6.25, and R^2^ of 0.941. In contrast, the baseline model yielded an MAE of 4.02, RMSE of 7.34, and R^2^ of 0.919. These results indicate that the proposed method demonstrates improved accuracy and robustness in spike count estimation, highlighting its potential for practical field applications.

**Figure 11:**
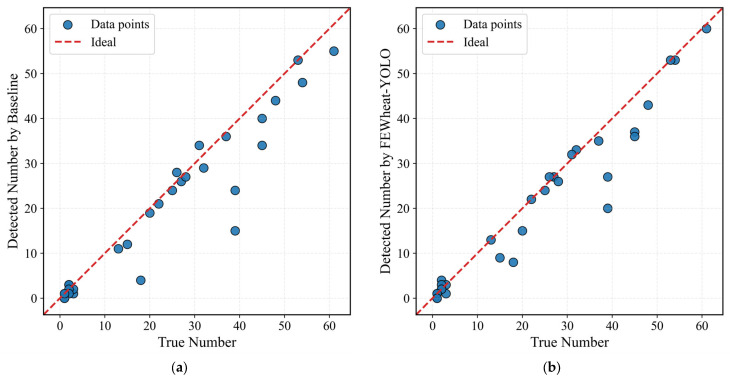
Correlation analysis between the predicted and true wheat spike counts for different models. (**a**) Baseline model (YOLOv11n); (**b**) Proposed FEWheat-YOLO model.

To further evaluate the performance of the models, we randomly selected 30 images and performed predictions using both the baseline model and our proposed FEWheat-YOLO model. Figure 11a,b present the correlation analysis between the predicted and true wheat spike counts. In the baseline model (Figure 11a), the correlation between the predicted and true values is relatively high when the true wheat spike counts are low. However, as the number of wheat spikes increases, particularly in cases of higher density or occlusion with the same image size, the scatter points become more dispersed, indicating lower prediction accuracy. In contrast, FEWheat-YOLO (Figure 11b) performs well when the wheat spike counts are low and demonstrates significantly better prediction accuracy when the true wheat spike count is high. The predicted values are closer to the true counts, especially in high-density regions, highlighting FEWheat-YOLO’s superior generalization ability and robustness.

In summary, FEWheat-YOLO outperforms the baseline model in wheat spike count estimation, particularly in high-density and occlusion scenarios, demonstrating better accuracy and stability. Compared to the baseline model, FEWheat-YOLO maintains high prediction accuracy across various scenarios, proving its superiority and practicality in the wheat spike counting task.

### 3.3. Ablation Study

Table 5 presents the ablation study results of each component in FEWheat-YOLO. To verify the effectiveness of each component in FEWheat-YOLO for wheat spike detection, we conducted a series of ablation experiments by gradually integrating individual modules into the baseline model, YOLOv11. All experiments were performed under identical environments and hyperparameters to ensure fairness.

Experimental results demonstrate that introducing the FEMANet module as the feature extraction backbone led to improvements of 0.23%, 0.4%, 0.4%, 0.1%, 0.2% in AP, AP@50, AP_s, AP_m, and AP_l respectively, with only a marginal increase of 0.5 GFLOPs in computational cost. Moreover, the number of parameters and model file size were reduced by 0.99 M and 1.8 MB, respectively.

Subsequently, the use of BiAFA-FPN as the feature fusion module resulted in a 0.5% improvement in AP_s. Notably, despite an increase of 0.4 GFLOPs in computational complexity, the number of parameters and model size decreased by 0.55 M and 1.1 MB, respectively.

Next, the introduction of ADown as the downsampling module led to reductions of 0.22 M in parameters, 1.1 GFLOPs in computation, and 0.4 MB in model size. Although there was a slight drop in accuracy metrics, this trade-off is considered acceptable given the significant reduction in computational cost.

Finally, the adoption of GSCDHead as the detection head resulted in improvements across all accuracy indicators: AP, AP@50, AP_s, AP_m, AP_l, and AR increased by 0.38%, 0.2%, 0.8%, 0.2%, and 0.3%, respectively. In addition, the number of parameters, computational cost, and model size were reduced by 0.15 M, 0.8 GFLOPs, and 0.3 MB, respectively.

In summary, the integration of FEMANet, BiAFA-FPN, ADown, and GSCDHead into FEWheat-YOLO yielded significant enhancements. The model achieved AP@50, AP_s, AP_m, AP_l, and AR scores of 51.11%, 89.8%, 18.1%, 50.5%, 61.2%, and 58.1%, respectively. Compared with the YOLOv11 baseline, these represent improvements of 0.53%, 0.7%, 0.7%, 0.4%, and 0.7%, respectively. Furthermore, the parameter count, computational complexity, and model size were reduced by 74%, 15.9%, and 69.2%, respectively. The design allows FEWheat-YOLO to achieve higher detection accuracy with a substantial reduction in parameter count and computational complexity, making it well-suited for deployment on devices with limited processing capability.

### 3.4. Backbone Network Comparison

To further validate the effectiveness of the proposed FEMANet as a backbone network for wheat spike detection, we adopt YOLOv11 as the baseline framework and replace its default backbone with several representative lightweight architectures. All experiments are conducted under identical training configurations and dataset settings to ensure fair comparison. The backbone networks involved in the comparison include FasterNet, Reversible Column Networks [63], ConvNeXt V2 [64], MobileNetV4 [65], StarNet [66], and the proposed FEMANet. The detection performance, model complexity, computational cost, inference speed, and model size of each network are summarized in Table 6.

Experimental results show that FEMANet exhibits superior performance across several key evaluation metrics. It achieves the highest overall Average Precision (AP = 50.80%) and Average Recall (AR = 57.8%) among all backbones, and maintains a balanced detection capability across small (AP_s = 17.8%), medium (AP_m = 50.2%), and large (AP_l = 61.1%) scale targets. Notably, FasterNet outperforms FEMANet slightly in some individual metrics, such as AP_m (51.5%), AP_l (61.6%), and inference speed (56 FPS). However, this comes at the cost of significantly higher resource consumption, with 3.90 M parameters, 9.2 GFLOPs, and a model size of 7.8 MB.

In contrast, FEMANet contains only 1.59 M parameters, requires 6.8 GFLOPs, and has a compact model size of 3.4 MB, demonstrating clear advantages in lightweight design. Although its inference speed (45 FPS) is slightly lower than that of MobileNetV4 and StarNet, FEMANet achieves a more favorable balance among detection accuracy, model compactness, and computational efficiency. Therefore, FEMANet is more suitable for deployment on resource-constrained edge devices and embedded platforms, making it a practical backbone choice for real-world agricultural applications involving wheat spike detection.

**Figure 12:**
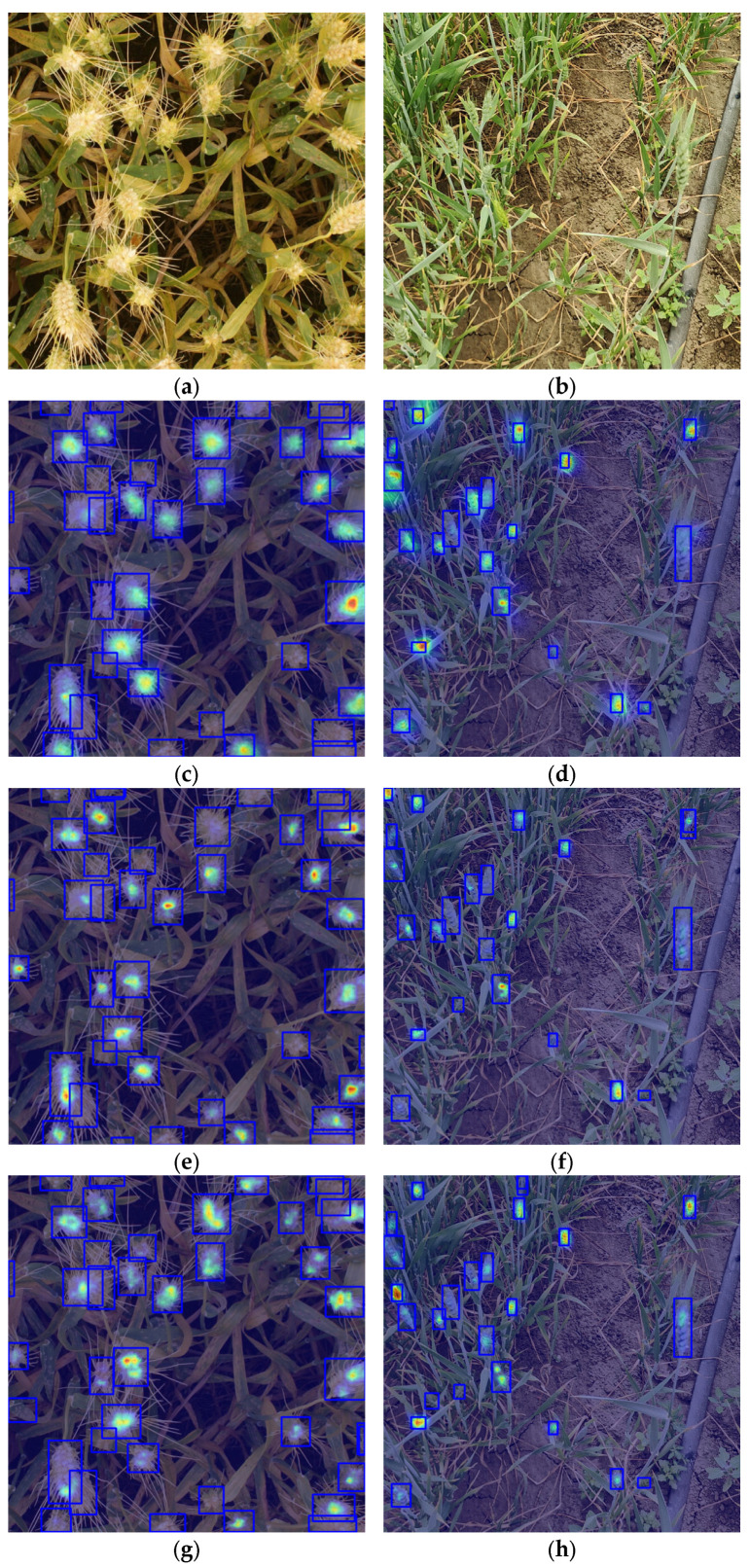

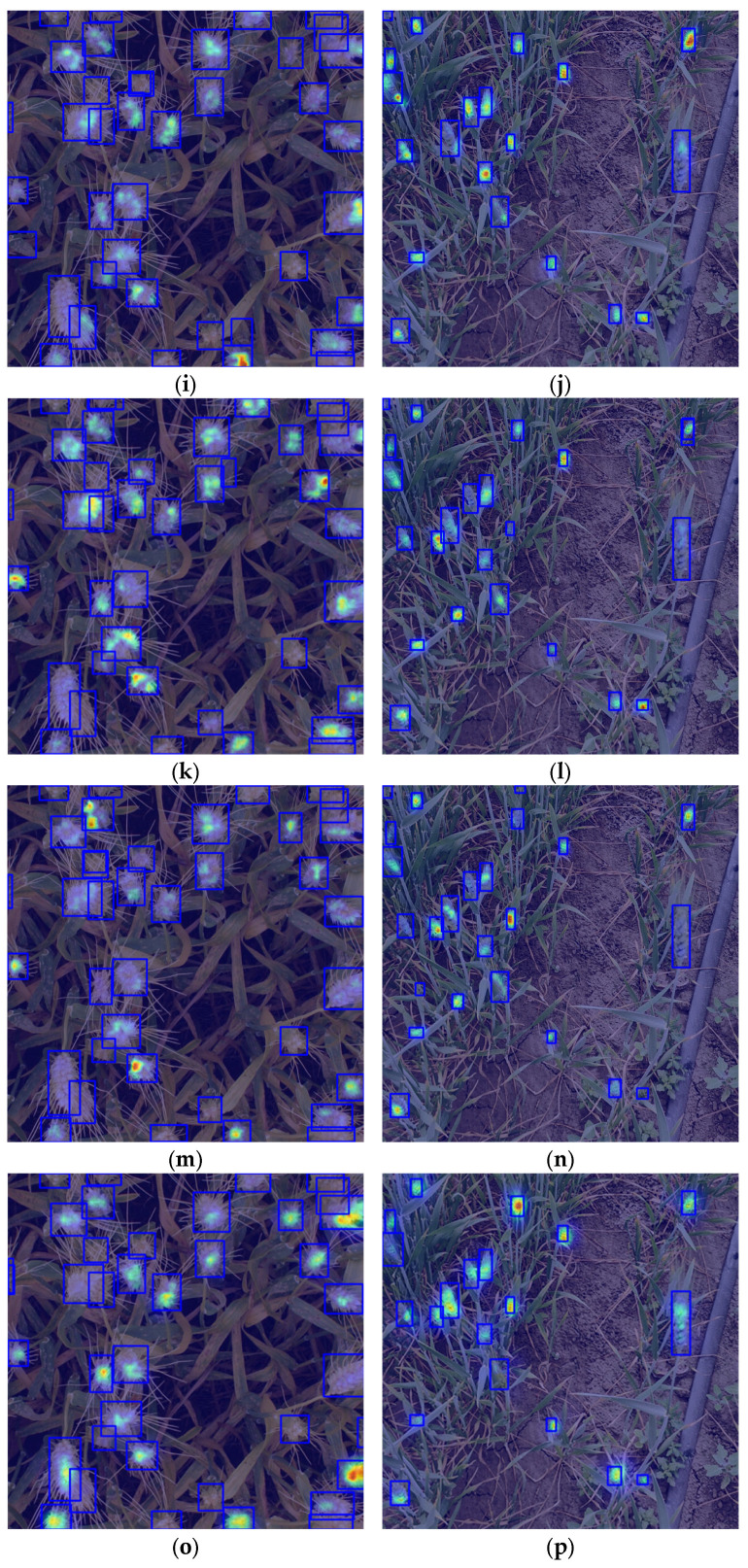
HiResCAM-based heatmap visualizations for different backbone networks. The left column shows the results for the public dataset, and the right column shows the results for the private dataset. (**a**,**b**) Original image; (**c**,**d**) YOLO11n (baseline); (**e**,**f**) FasterNet; (**g**,**h**) Reversible Column Networks; (**i**,**j**) ConvNeXt V2; (**k**,**l**) MobileNetV4; (**m**,**n**) StarNet; (**o**,**p**) The proposed FEMANet. The red regions indicate the model’s response intensity to wheat spike areas. The blue boxes represent the predicted wheat spikes, and the color variations, with warmer colors indicating stronger activations.

To further investigate the attention regions and feature response mechanisms of different lightweight backbone networks in wheat spike detection, this study adopts HiResCAM (High-Resolution Class Activation Mapping) [67] to visualize the inference process. Compared with conventional CAM [68] and Grad-CAM [69] methods, HiResCAM generates activation maps with higher spatial resolution and sharper edges, making it particularly suitable for the interpretation and discrimination of fine-grained targets such as densely distributed wheat spikes. Figure 12 presents the heatmap visualizations of various backbone networks, using representative samples from the public dataset (left column) and the private dataset (right column). As shown in Figure 12a,b, the original images exhibit densely distributed wheat spikes and complex backgrounds, which present significant challenges for detection. The tightly clustered wheat spikes, along with the presence of various background elements such as soil, other vegetation, and irrigation systems, make it difficult for models to accurately focus on the wheat spike regions. As shown in Figure 12c,d, the YOLO11n baseline exhibits dispersed activation regions, with limited focus on actual wheat spike areas. Similarly, models such as FasterNet (Figure 12e,f), Reversible Column Networks (RevCol) (Figure 12g,h), ConvNeXt V2 (Figure 12i,j), MobileNetV4 (Figure 12k,l), and StarNet (Figure 12m,n) show shifted or incomplete responses under complex backgrounds or occlusion conditions. In contrast, the proposed FEMANet (Figure 12o,p) consistently demonstrates clear, well-localized activation regions across both datasets, effectively covering wheat spike targets. These results indicate superior spatial perception and generalization performance.

### 3.5. Performance Comparison of Feature Pyramid Networks

The results of comparing feature fusion modules for wheat spike detection are summarized in Table 7, where we evaluated four representative FPN-based structures: BiFPN [53], MAFPN [70], AFPN [71], and the proposed BiAFA-FPN, using FEMANet as the backbone. All experiments were performed under identical training settings to ensure fairness.

In terms of overall detection accuracy, MAFPN achieved the highest AP of 50.99% and AR of 58.0%, slightly outperforming the other methods. However, it comes with a higher parameter count (1.68 M), larger computational complexity (7.5 GFLOPs), and slower inference speed (45 FPS), which limit its suitability for real-time applications.

AFPN, on the other hand, offered the smallest model size (2.3 MB) and a relatively low parameter count (0.93 M), but showed the weakest performance in both accuracy (AP = 49.94%) and inference speed (37 FPS), making it less practical for deployment.

BiFPN presented a well-balanced trade-off with the lowest parameter count (0.88 M) and smallest model file (2.1 MB), while still achieving solid results in AP (50.89%) and AR (57.7%), demonstrating its efficiency in compact model design.

In contrast, the proposed BiAFA-FPN strikes a more favorable balance across accuracy, inference speed, and model complexity. It achieved AP and AR scores of 50.82% and 57.9%, respectively—comparable to MAFPN and BiFPN—but significantly outperformed all other modules in inference speed, reaching 56 FPS, the highest among the compared methods. While its small object detection accuracy (AP_s = 18.3%) was slightly lower than that of MAFPN (18.6%), it surpassed BiFPN (17.8%), indicating stronger representational capacity for dense and small-scale targets.

Additionally, BiAFA-FPN maintained a moderate model size (1.04 M parameters, 2.3 MB) and computational cost (7.2 GFLOPs), remaining within a deployable range.

In summary, BiAFA-FPN delivers an excellent balance between detection accuracy, inference speed, and model compactness, making it particularly well-suited for agricultural vision tasks that demand real-time performance, small object recognition, and deployment efficiency.

### 3.6. Ablation Study on Feature Fusion Strategies

To compare the performance of different feature fusion methods, we conducted experiments under identical conditions using a YOLOv11n model integrated with FEMANet, BiAFA-FPN, and GSCDHead. Specifically, BiFPN [53], SDI [72], Adaptive and Weight [73], and Concat were employed as the feature fusion modules. The corresponding performance evaluation results are summarized in Table 8.

As shown in Table 8, the model using Concat as the feature fusion strategy achieved the best overall performance across all evaluation metrics. Specifically, compared with SDI, Adaptive, BiFPN, and Weight, Concat improved AP by 1.39%, 1.06%, 0.5%, and 0.6%, respectively, and enhanced AR by 1.1%, 0.6%, 0.5%, and 0.6%, respectively. For the more challenging task of detecting small-scale wheat spikes, Concat improved AP_s by 0.8%, 0.6%, 0.8%, and 1.1%, respectively. It also achieved gains in AP_m by 0.9%, 0.5%, 0.4%, and 0.6%, and in AP_l by 2.2%, 0.7%, 0.1%, and 0.2%, respectively.

In terms of computational efficiency, Concat reduced GFLOPs by 0.3, 0.2, and 0.2 compared to SDI, Adaptive, and Weight, respectively. Although BiFPN shared a similar model file size with Concat (due to rounding to one decimal place), it had 0.2 fewer GFLOPs and slightly fewer parameters. However, its detection performance was inferior, with decreases of 0.39%, 0.8%, 0.4%, 0.1%, and 0.5% in AP, AP@50, AP_s, AP_m, and AR, respectively.

In conclusion, Concat achieves the highest detection accuracy while maintaining low computational and storage costs, and is therefore adopted as the feature fusion method in the BiAFA-FPN module.

### 3.7. Comparison of Downsampling Methods

To evaluate the performance of ADown, we adopted YOLOv11n integrated with FEMANet, BiAFA-FPN, and GSCDHead as the baseline model. Under identical experimental settings, the original downsampling module (Conv) was replaced by LDConv [74], HWD [75], SRFD [76], PSConv [77], and ADown, respectively. The evaluation results are summarized in Table 9.

As shown in Table 9, employing ADown as the downsampling module yields outstanding detection performance, with AP, AP@50, AP_s, AP_m, AP_l, and AR reaching 51.11%, 89.8%, 18.1%, 50.5%, 61.2%, and 58.1%, respectively. Among these, AP, AP@50, AP_s, AP_m, and AR are the highest across all compared models.

Although SRFD achieves the same AP_m (50.5%) as ADown, it comes at a significantly higher cost—its parameter count increases by 0.21 M, GFLOPs rise by 2.9, and model size grows by 0.6 MB.

PSConv shares the same model size as ADown (1.6 MB), but its parameter count is 0.1 M higher and computational cost is 0.2 GFLOPs more. However, these additional resources fail to translate into performance gains. Compared with ADown, PSConv yields lower scores across all metrics: AP, AP@50, AP_s, AP_m, AP_l, and AR decrease by 0.63%, 0.3%, 0.1%, 0.5%, 0.5%, and 0.5%, respectively.

In conclusion, ADown not only reduces computational and storage costs but also contributes to accuracy improvement. Therefore, it is selected as the downsampling module in FEWheat-YOLO.

### 3.8. Deployment of FEWheat-YOLO on Raspberry Pi

In this study, we exported FEWheat-YOLO into various formats, including PyTorch, ONNX, NCNN, OpenVINO, and Paddle, for deployment on the Raspberry Pi. The best deployment format was chosen based on the fastest inference speed observed during model evaluation, as shown in Table 10.

The table demonstrates the deployment performance of different models on the Raspberry Pi, with a comparison of inference time (latency), FPS (frames per second), CPU usage, and memory usage. The ONNX model performs the best, offering the shortest inference time (0.6 s) and the highest FPS (1.7), making it ideal for real-time applications, despite its relatively high CPU usage (95%). On the other hand, PyTorch has a longer inference time (1.7 s) and the lowest FPS (0.6), but it uses less memory. NCNN and OpenVINO strike a good balance with moderate inference speeds and FPS, although they have higher memory usage (around 86%). Paddle performs the worst, with the longest inference time (1.8 s), the lowest FPS (0.5), and the highest memory usage (91.7%).

In conclusion, the ONNX model is the best choice for real-time deployment. Based on our findings, we recommend converting the model to ONNX format for deployment on the Raspberry Pi.

## 4. Discussion

### 4.1. Background and Related Work

In wheat breeding and yield estimation, accurate spike detection and counting are essential for informed decision-making and effective field management [78,79]. Consequently, wheat spike detection has attracted significant research attention. For example, Zang et al. [80] improved the DM-Count framework and proposed the DMseg-Count model, incorporating a localized segmentation branch and a dedicated fusion mechanism to integrate global and local contextual information of wheat spikes. This model achieved promising detection results with MAE and RMSE of 5.79 and 7.54, respectively, in the counting task. However, its large model size (92.23 M parameters, 23.88 GFLOPs, 24.18 MB) entails high computational and storage requirements. Similarly, Yue et al. [81] developed a relatively lightweight model, CML-RTDETR, by optimizing the backbone network and redesigning a multi-scale feature enhancement pyramid, achieving 90.5% detection accuracy. After pruning, the model size was reduced to 11.03 M parameters and 37.8 GFLOPs, with an inference speed of 73 FPS, but it still required high-performance hardware, limiting deployment in resource-constrained environments [82,83,84].

### 4.2. Advances in Lightweight Wheat Spike Detection

To address resource limitations, some researchers have explored lightweight wheat spike detection and counting methods. For instance, Guan et al. [21] improved YOLOv10 by integrating BiFPN, SEAM, and GCNet, achieving 93.69% accuracy and, in the counting task, R^2^ of 0.96, MAE of 3.57, and RMSE of 4.09, with a model size of only 3.0 M parameters. Qiu et al. [85] built a multi-growth-stage wheat spike dataset and employed lightweight backbones such as MobileNet and ShuffleNet, combined with DWDown and LightDetect for improved downsampling and detection heads, proposing LGWheatNet with 1.70 M parameters, 5.0 GFLOPs, and 95.6% accuracy. While these lightweight designs perform well on resource-limited devices, further improvements are needed to maintain high precision while reducing parameters and computation even further.

### 4.3. Proposed Method: FEWheat-YOLO

To overcome these challenges, this study introduces FEWheat-YOLO, a lightweight detection model built upon the YOLOv11n framework, incorporating FEMANet, BiAFA-FPN, ADown, and GSCDHead modules. These innovations effectively reduce parameters and computational complexity while significantly improving wheat spike detection accuracy. On the HWHD dataset, FEWheat-YOLO achieved AP 51.11%, AP@50 89.8%, AP_s 18.1%, AP_m 50.5%, AP_l 61.2%, and AR 58.1%. The COCO evaluation system allows fine-grained assessment across target scales, with AP_s in particular reflecting the model’s capability for small-object detection—critical for accurate counting and yield estimation in complex field conditions. Remarkably, this performance was achieved with only 0.67 M parameters, 5.3 GFLOPs, and 1.6 MB model size.

### 4.4. Comparative Analysis with Existing Methods

Compared to existing methods—DMseg-Count [75] (92.23 M parameters, 23.88 GFLOPs, 24.18 MB), CML-RTDETR [76] (11.03 M, 37.8 GFLOPs), improved YOLOv10 [80] (3.0 M), and LGWheatNet [81] (1.70 M, 5.0 GFLOPs)—FEWheat-YOLO achieves the smallest parameter count and model size, with competitive or superior detection performance. It should be noted that for some methods (e.g., improved YOLOv10), FLOPs and model size were not fully reported in the original papers, so comparisons are based on publicly available data.

Furthermore, experiments comparing YOLOv5n through YOLOv13n, as well as SSDLite-MobileNetV2, Faster R-CNN, and RT-DETR-R18, show that FEWheat-YOLO achieved the best AP, AP@50, and AP_m, and tied with RT-DETR-R18 on AP_s. However, its parameters, computation, and model size were only 3.4%, 9.3%, and 4.2% of those of RT-DETR-R18, respectively. This indicates that FEWheat-YOLO delivers accuracy gains while greatly reducing storage and computation demands, enabling deployment on agricultural edge devices.

### 4.5. Practical Applicability

Benefiting from the computational capability of the RTX 3070 Ti GPU (NVIDIA, Santa Clara, CA, USA), FEWheat-YOLO achieves a real-time inference speed of 54 FPS, meeting the practical demand (≥30 FPS) in agricultural applications. Moreover, due to its lightweight architecture, the model is also deployable on resource-constrained devices such as RTX 2060, RTX 1050, and CPU-only platforms, making it suitable for edge computing and embedded environments.

### 4.6. Limitations and Future Work

Despite its advantages, FEWheat-YOLO has some limitations. First, although we augmented the GWHD dataset with a custom dataset to improve generalization, data collection was limited to the “Xin Chun 40” wheat variety in Alar City, lacking samples from diverse varieties and regions. Factors such as cultivar type, growing environment, and climate affect phenotypic traits, limiting the model’s generalization. Additionally, our dataset was primarily collected under sunny conditions in Xinjiang, where cloudy and rainy days are rare, potentially reducing performance in adverse weather. Future work will expand the dataset to include a wider range of climatic conditions, regions, and capture devices—including UAVs, digital cameras, near-infrared, and thermal imaging systems—to enhance adaptability.

Second, current experiments were conducted on a desktop GPU, without validation on embedded platforms such as Jetson or Raspberry Pi. While FEWheat-YOLO’s lightweight design theoretically supports low-power deployment, real-world performance in terms of stability, power consumption, and latency remains to be verified. Future work will involve embedded platform testing to ensure compatibility with real-time agricultural automation requirements.

Third, although optimized for wheat spikes, FEWheat-YOLO may not perform equally well on other panicle crops such as maize or rice. Retraining or fine-tuning will likely be necessary. Expanding training to multi-crop datasets or employing transfer learning could enhance cross-crop adaptability and improve applicability in diverse agricultural settings.

Moreover, the complexity of field environments—with weeds, other crops, and significant occlusion—can challenge target discrimination, leading to missed or false detections. Integrating advanced background separation or local enhancement techniques, such as deep-learning-based segmentation or regional attention mechanisms, may improve robustness under such conditions.

Finally, while FEWheat-YOLO achieves 54 FPS on 1024 × 1024 images, higher resolutions or more complex scenes may cause performance bottlenecks, especially in high-speed agricultural systems. Model compression techniques such as quantization, pruning, and knowledge distillation, along with hardware acceleration, will be explored to further boost inference efficiency. Since FEWheat-YOLO is currently designed for static image detection, incorporating multi-object tracking (e.g., DeepSORT, FairMOT) and temporal reasoning could significantly improve performance in video-based spike counting, enabling more accurate real-time yield estimation.

## 5. Conclusions

This study introduces FEWheat-YOLO, an ultra-lightweight wheat spike detection model, which integrates the FEMANet, BiAFA-FPN, ADown, and GSCDHead modules into the YOLOv11n framework. The model significantly enhances detection accuracy while reducing computational complexity and storage requirements.

1. Model Performance

FEWheat-YOLO demonstrates exceptional performance on a mixed dataset, achieving 51.11% AP, 89.8% AP@50, 18.1% AP_s, and 58.1% AR. The model has 0.67 M parameters, a 1.6 MB size, and 5.3 GFLOPs computational complexity, showcasing its efficiency in resource-constrained environments.

2. Comparison with Existing Methods

Compared to various state-of-the-art models, including the YOLO family, Faster R-CNN, SSDLite-MobileNetV2, and RT-DETR-R18, FEWheat-YOLO outperforms in AP, AP@50, and AP_m, and excels in AP_l and AR. Notably, its parameter count, computation, and model size are only 3.4%, 9.3%, and 4.2% of RT-DETR-R18, respectively, significantly reducing storage and computational demands while maintaining high accuracy.

3. Practical Applications

With its lightweight design, FEWheat-YOLO demonstrates the capability of achieving real-time operation on the Raspberry Pi, offering an efficient and practical solution for agricultural automation and intelligent sensing in resource-constrained environments.

Future research will focus on the following directions:

1. Dataset Expansion: Expanding the dataset to cover more crop varieties and different climatic conditions to improve the model’s generalization and adaptability.
2. Cross-Crop Generalization: Enhancing the model’s applicability to different crops (e.g., maize, rice) to ensure its effectiveness in diverse agricultural environments.
3. Embedded Platform Adaptation: Optimizing the model for embedded platforms (e.g., Jetson, Raspberry Pi), evaluating its stability, performance, and practicality on low-power devices, and further conducting field validation to confirm its effectiveness in real-world agricultural environments.
4. Video Inference and Multi-Object Tracking: Integrating video-based inference and multi-object tracking techniques to improve real-time detection accuracy and stability in dynamic environments.

## Figures and Tables

**Table 1:** Distribution of images in the training, validation, and test sets for each dataset.

Dataset Name	Training	Validation	Test	Total
GWHD2021	4495	1284	643	6422
custom	1540	440	220	2200
HWHD	6035	1724	863	8622

**Table 2:** Training hyperparameters for the WheatFE-YOLO model, including batch size, learning rate, number of epochs, input image size, optimizer type, momentum, and IoU threshold for object detection.

Set of Parameters	Value or Name
Batch size	8
Learning rate	0.01
Epoch	200
Image resize	640 × 640
Optimizer	SGD
Momentum	0.937
IoU-thres	0.55

**Table 3:** Performance comparison of the proposed method and representative object detectors on the HWHD dataset. The bolded values represent the optimal indicators.

Model	AP/%	AP@50 /%	AP_s/%	AP_m/%	AP_l/%	AR/%	Parameters	GFLOPS	FPS	Model Size /MB
YOLOv5-n	49.98	88.7	16.2	49.3	61.0	57.0	2.50 × 10^6^	7.1	110	5.0
YOLOv6-n	49.45	88.5	16.2	49.0	60.0	56.6	4.23 × 10^6^	11.8	111	8.3
YOLOv7-Tiny	49.51	89.4	17.5	49.0	59.8	56.1	6.02 × 10^6^	13.2	**136**	11.7
YOLOv8-n	50.47	89.0	17.6	49.8	61.3	57.4	3.01 × 10^6^	8.1	114	6.0
YOLOv9-t	49.95	89.1	16.7	49.2	61.1	56.7	2.62 × 10^6^	10.7	34	5.8
YOLOv10-n	50.56	88.5	16.9	50.1	61.0	**58.3**	2.27 × 10^6^	6.5	96	5.5
YOLOv12-n	50.31	88.9	16.5	49.8	60.9	57.2	2.51 × 10^6^	5.8	52	5.2
YOLOv13-n	49.80	88.7	17.1	49.4	59.5	56.8	2.45 × 10^6^	6.2	40	5.2
Faster R-CNN	44.60	83.9	9.5	44.0	56.6	51.1	4.13 × 10^7^	90.9	33	78.8
SSDLite-MobileNetV2	38.20	80.0	4.4	37.5	49.7	47.4	3.03 × 10^6^	**2.8**	46	5.8
RT-DETR-R18	50.73	89.1	**18.1**	50.1	**61.8**	57.3	1.99 × 10^7^	56.9	92	38
FEWheat-YOLO	**51.11**	**89.8**	**18.1**	**50.5**	61.2	58.1	**6.73** **×** **10^5^**	5.3	54	**1.6**

**Table 4:** Comparison between the baseline algorithm and FEWheat-YOLO on wheat spike count estimation metrics. Bold numbers indicate the best performance. The bolded values represent the optimal indicators.

Method	MAE	RMSE	R^2^
Baseline	4.02	7.34	0.919
Ours	**3.46**	**6.25**	**0.941**

**Table 5:** Ablation study results of FEWheat-YOLO.The symbol √ indicates that the corresponding module was used, while the symbol × denotes that the module was not used.

FEMANet	BiAFA-FPN	ADown	GSCDHead	AP/%	AP@50/%	AP_s/%	AP_m/%	AP_l/%	AR/%	Parameters	GFLOPS	FPS	Model Size (MB)
×	×	×	×	50.57	89.1	17.4	50.1	60.9	57.4	2.58 × 10^6^	6.3	84	5.2
√	×	×	×	50.80	89.5	17.8	50.2	61.1	57.8	1.59 × 10^6^	6.8	45	3.4
√	√	×	×	50.82	89.6	18.3	50.3	61.0	57.9	1.04 × 10^6^	7.2	56	2.3
√	√	√	×	50.72	89.6	17.3	50.3	60.9	57.8	8.2 × 10^5^	6.1	54	1.9
√	√	√	√	51.11	89.8	18.1	50.5	61.2	58.1	6.7 × 10^5^	5.3	54	1.6

**Table 6:** Comparison of different backbone networks integrated into YOLO11. The bolded values represent the optimal indicators.

Backbone Network	AP/%	AP@50/%	AP_s/%	AP_m/%	AP_l/%	AR/%	Parameters	GFLOPS	FPS	Model Size (MB)
FasterNet	50.52	89.3	**17.8**	**51.5**	**61.6**	**57.8**	3.90 × 10^6^	9.2	56	7.8
RevCol	48.80	87.3	14.9	48.2	59.8	55.9	2.09 × 10^6^	**4.9**	39	4.5
ConvNeXt V2	50.60	89.1	17.6	50.1	61.4	57.5	5.39 × 10^6^	12.5	60	10.6
MobileNetV4	49.08	88.2	15.4	48.6	59.7	56.3	5.43 × 10^6^	21.0	**65**	10.7
StarNet	49.61	88.5	16.3	49.0	60.5	56.7	1.94 × 10^6^	5.0	64	4.0
FEMANet (ours)	**50.80**	**89.5**	**17.8**	50.2	61.1	**57.8**	**1.59** **× 10^6^**	6.8	45	**3.4**

**Table 7:** Detection performance and efficiency comparison of the proposed BiAFA-FPN and other representative FPN. architectures under the FEMANet backbone. The bolded values represent the optimal indicators.

Neck Structure	AP/%	AP@50/%	AP_s/%	AP_m/%	AP_l/%	AR/%	Parameters	GFLOPS	FPS	Model File Size (MB)
MAFPN	**50.99**	**89.8**	**18.6**	**50.6**	**61.2**	**58.0**	1.68 × 10^6^	7.5	45	3.6
AFPN	49.94	88.8	17.2	49.5	60.0	57.2	9.31 × 10^5^	**6.1**	37	2.3
BiFPN	50.89	89.7	17.8	50.4	61.1	57.7	**8.84** **×** **10^5^**	6.4	41	**2.1**
BiAFA-FPN (ours)	50.82	89.6	18.3	50.3	61.0	57.9	1.04 × 10^6^	7.2	**56**	2.3

**Table 8:** Performance comparison of different feature fusion methods integrated into the BiAFA-FPN. The bolded values represent the optimal indicators.

Feature Fusion Method	AP/%	AP@50/%	AP_s/%	AP_m/%	AP_l/%	AR/%	Parameters	GFLOPS	FPS	Model Size (MB)
SDI	49.72	88.9	17.3	49.6	59.0	57.0	7.0 × 10^5^	5.6	41	1.8 MB
Adaptive	50.05	88.9	17.5	50.0	60.5	57.5	7.1 × 10^5^	5.5	39	1.8 MB
BiFPN	50.61	89.6	17.3	50.1	61.1	57.6	**6.5** **×** **10^5^**	**5.1**	42	**1.6 MB**
Weight	50.51	89.5	17.0	49.9	61.0	57.5	7.1 × 10^5^	5.5	42	1.8 MB
Concat (ours)	**51.11**	**89.8**	**18.1**	**50.5**	**61.2**	**58.1**	**6.7 × 10^5^**	**5.3**	**54**	**1.6 MB**

**Table 9:** Comparison of different downsampling methods under the same detection framework. The original Conv module was replaced by LDConv, HWD, SRFD, PSConv, and ADown, respectively. The bolded values represent the optimal indicators.

Downsampling Module	AP/%	AP@50/%	AP_s/%	AP_m/%	AP_l/%	AR/%	Parameters	GFLOPS	FPS	Model Size (MB)
LDConv	50.51	89.4	17.6	50.0	60.9	57.6	7.1 × 10^5^	6.2	22	1.8
HWD	50.49	89.3	17.5	50.1	60.6	57.5	7.2 × 10^5^	6.1	41	1.8
SRFD	50.86	89.7	17.4	**50.5**	61.0	57.9	8.8 × 10^5^	8.2	42	2.2
PSConv	50.48	89.5	18.0	50.0	60.7	57.6	7.7 × 10^5^	5.5	49	**1.6**
ADown (ours)	**51.11**	**89.8**	**18.1**	**50.5**	**61.2**	**58.1**	**6.7** **× 10^5^**	**5.3**	**54**	**1.6**

**Table 10:** Performance Comparison of Model Deployment on Raspberry Pi.

Deployment Format	Inference Time per Images (s)	FPS	CPU Usage (%)	Memory Usage (%)
ONNX	0.6	1.7	95.0	42.5
PyTorch	1.7	0.6	97.6	23.1
NCNN	0.8	1.3	94.6	86.8
OpenVINO	0.8	1.3	94.6	86.8
Paddle	1.8	0.5	79.7	91.7

## Data Availability

The public part of the dataset used in this study is available from the Global Wheat Head Detection (GWHD2021) dataset at https://www.kaggle.com/competitions/global-wheat-detection, accessed on 30 September 2025. The remaining part of the dataset is private and cannot be shared due to institutional or privacy restrictions. Requests for access to the private dataset may be directed to the corresponding author.

## Abbreviations

The following abbreviations are used in this manuscript:

YOLO	You Only Look Once
FEMANet	Faster-Enhanced Mixed Aggregation Network with Efficient Multi-scale Attention
BiAFA-FPN	Bidirectional Asymmetric Feature Aggregation Feature Pyramid Network
ADown	Adaptive Downsampling
GSCDHead	Grouped Shared Convolution Detection Head
CNNs	Convolutional Neural Networks
AP	Average Precision
AR	Average Recall
FPS	Frames Per Second
COCO	Common Objects in Context
MAE	Mean Absolute Error
R^2^	Coefficient of Determination
RMSE	Root Mean Square Error
GWHD2021	Global Wheat Head Detection 2021
RPN	Region Proposal Network
ROI	Region of Interest
SSD	Single Shot Multibox Detector
RT-DETR	Real-Time Detection Transformer
DeepSORT	Deep Learning-based SORT
HWHD	Hybrid Wheat Head Detection Dataset
IoU	Intersection over Union
FLOPs	Floating-point Operations
Params	Number of trainable parameters
EMA	Efficient Multi-scale Attention
DFL	Distribution Focal Loss
FPN	Feature Pyramid Network
